# Complications of Tranexamic Acid in Orthopedic Lower Limb Surgery: A Meta-Analysis of Randomized Controlled Trials

**DOI:** 10.1155/2021/6961540

**Published:** 2021-01-16

**Authors:** Davide Reale, Luca Andriolo, Safa Gursoy, Murat Bozkurt, Giuseppe Filardo, Stefano Zaffagnini

**Affiliations:** ^1^Clinica Ortopedica e Traumatologica II, IRCCS Istituto Ortopedico Rizzoli, Bologna, Italy; ^2^Department of Orthopaedics and Traumatology, Ankara Yildirim Beyazit University, Ankara, Turkey; ^3^Applied and Translational Research Center, IRCCS Istituto Ortopedico Rizzoli, Bologna, Italy; ^4^Facoltà di Scienze Biomediche, Università della Svizzera Italiana, Lugano, Switzerland

## Abstract

**Objective:**

Tranexamic acid (TXA) is increasingly used in orthopedic surgery to reduce blood loss; however, there are concerns about the risk of venous thromboembolic (VTE) complications. The aim of this study was to evaluate TXA safety in patients undergoing lower limb orthopedic surgical procedures.

**Design:**

A meta-analysis was performed on the PubMed, Web of Science, and Cochrane Library databases in January 2020 using the following string (Tranexamic acid) AND ((knee) OR (hip) OR (ankle) OR (lower limb)) to identify RCTs about TXA use in patients undergoing every kind of lower limb surgical orthopedic procedures, with IV, IA, or oral administration, and compared with a control arm to quantify the VTE complication rates.

**Results:**

A total of 140 articles documenting 9,067 patients receiving TXA were identified. Specifically, 82 studies focused on TKA, 41 on THA, and 17 on other surgeries, including anterior cruciate ligament reconstruction, intertrochanteric fractures, and meniscectomies. The intravenous TXA administration protocol was studied in 111 articles, the intra-articular in 45, and the oral one in 7 articles. No differences in terms of thromboembolic complications were detected between the TXA and control groups neither in the overall population (2.4% and 2.8%, respectively) nor in any subgroup based on the surgical procedure and TXA administration route.

**Conclusions:**

There is an increasing interest in TXA use, which has been recently broadened from the most common joint replacement procedures to the other types of surgeries. Overall, TXA did not increase the risk of VTE complications, regardless of the administration route, thus supporting the safety of using TXA for lower limb orthopedic surgical procedures.

## 1. Introduction

Lower limb procedures represent the majority of orthopedic surgeries, including joint arthroplasties, sport medicine treatments, and fracture osteosynthesis, with a high rate of over 500 per 100,000 population every year, increasing over time [1, 2]. Among these, total hip arthroplasty (THA) and total knee arthroplasty (TKA) are the most commonly performed. Albeit being successful procedures routinely performed in the clinical practice, they are frequently encumbered by complications [3]. In particular, joint arthroplasties are often associated with a significant amount of postoperative blood loss, ranging from 800 to 1,800 ml in primary TKA or THA, with up to 25% and 37% of patients, respectively, requiring blood transfusion for postoperative anemia [4, 5]. Allogenic blood transfusions are financial burdens, and even more importantly, they are associated with an unneglectable risk of serious complications, including infection, immunosuppression, and cardiovascular dysfunction [6], resulting in potentially life-threatening effects on patients [7]. Various strategies have been attempted to minimize blood loss and the need for blood transfusion [8], and to this aim, the use of hemostatic agents, in particular of tranexamic acid (TXA), has recently widely increased in orthopedic lower limb surgery.

TXA is a synthetic antifibrinolytic agent that competitively blocks the lysine binding sites on plasminogen, thereby slowing the conversion of plasminogen to plasmin, thus preventing fibrin clot degradation [9, 10]. A large amount of randomized controlled trials (RCTs) and high-level evidence [11–14] converges in showing that TXA, applied either through systemic or local administration, is effective in reducing blood loss and subsequent transfusions in replacement procedures, as well as in other kinds of lower limb surgeries, including sport medicine procedures and fracture treatment [15, 16]. However, there are still concerns about the risk of increasing venous thromboembolic (VTE) complications, such as deep venous thrombosis (DVT) or pulmonary embolisms (PE) [17, 18]. In this light, it is of paramount importance to understand if the scientific high-level literature evidence supports the safety of TXA for the different orthopedic applications.

Thus, the aim of this study was to evaluate, through a meta-analysis of RCTs, the safety of TXA used in patients undergoing lower limb orthopedic surgical procedures, including joint arthroplasty, fracture treatment, and sport injuries.

## 2. Material and Methods

### 2.1. Search Strategy and Article Selection

**Figure 1:**
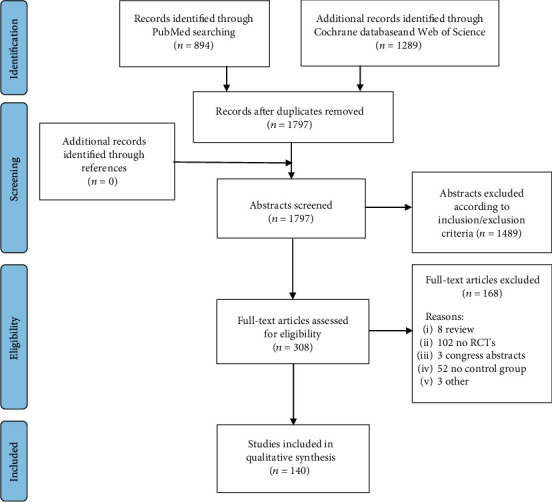
PRISMA (Preferred Reporting Items for Systematic Reviews and Meta-Analyses) flowchart of the study selection process.

A meta-analysis was performed based on the literature evidence about the thromboembolic complications of TXA in patients undergoing orthopedic lower limb surgery. The search was conducted on the PubMed, Web of Science, and Cochrane Library databases on January 9, 2020, using the following parameters: (Tranexamic acid) AND ((knee) OR (hip) OR (ankle) OR (lower limb)). The guidelines for Preferred Reporting Items for Systematic Reviews and Meta-Analyses (PRISMA) were used [19]. A flowchart of the study selection for the quantitative data synthesis is reported in Figure 1.

The screening process and analysis were conducted separately by 2 independent observers (DR and SG). In the first step, the articles were screened by title and abstract. The following inclusion criteria for relevant articles were used during the initial screening of titles and abstracts: RCTs, written in the English language, and about TXA use in patients undergoing every kind of lower limb surgical orthopedic procedure. Exclusion criteria were articles written in other languages, preclinical studies, studies of a different design than RCT, and reviews. No exclusions were made regarding the administration route (intra-articular (IA), intravenous (IV), and oral), dose (dosage amount and bolus versus continuous infusion), and timing of administration (preoperative, intraoperative, or postoperative).

In the second step, the full texts of the selected articles were retrieved and screened, with the further exclusion of RCTs without at least one treatment group receiving TXA and a control group, either receiving placebo or not. For example, surveys that had multiple treatment arms with other antifibrinolytic agents such as epsilon aminocaproic acid (EACA) were eligible, but only if they also analyzed separately one treatment arm with TXA and one control arm, with only TXA and control groups being included in the analysis. Moreover, studies not addressing the occurrence of VTE complications were excluded. No limitations were set based on the postoperative VTE chemoprophylaxis or on the method of screening or diagnosis for VTE used in each study. Reference lists from the selected papers were also screened, and all selected studies were included in the quantitative data synthesis.

### 2.2. Data Extraction, Outcome Measurement, and Statistical Analysis

The following information on trial methodology and included patients were extracted and collected in a database with the consensus of the two observers: publication year, study design, number of patients included, type of surgery, route of administration of TXA, and number of VTE complications to the purpose of this study. Accordingly, a meta-analysis on the VTE complication rate of the studies included was performed.

The statistical analysis and the forest plot were carried out according to Neyeloff et al. [20] using Microsoft Excel. The dichotomous outcome was a rare event, and most of the articles compared groups of similar dimensions and showed zero events, so Peto's method (Yusuf 1985 and Bradburn 2007) was used to provide a pooled odds ratio; the reason for the choice was that Peto's method does not need the corrections for zero cell counts. The statistical significance was based on the *z* statistics of Peto's estimation of the pooled odds ratio. *p* value of 0.05 was used as the level of statistical significance.

## 3. Results

**Figure 2:**
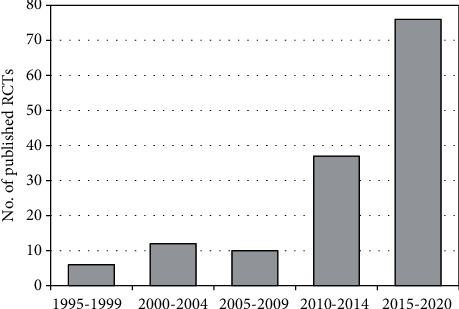
Publications' trend of RCTs about tranexamic acid in orthopedic lower limb surgery from 1995 to 2020.

The database search retrieved 1,797 records; out of these, 140 articles [11, 12, 15, 16, 21–157] were eligible for the meta-analysis of VTE (Figure 1). The publication trend showed that the majority of these RCTs were published in the last five years: 46% from 1996 to 2015 and 54% after 2015 (Figure 2).

Overall, 9,067 patients received TXA while 6,592 were included in the control groups. With regard to the type of surgery, 82 studies [21–24, 26–28, 30, 31, 34–38, 40, 45, 47, 49–51, 55–58, 60, 61, 63, 65, 66, 70, 72, 73, 75, 76, 78–82, 84, 85, 87, 88, 90, 92, 93, 96, 98, 99, 104–111, 113, 114, 117–119, 121, 123, 124, 126–128, 130, 134–137, 140–142, 146, 147, 151, 153, 154, 156] (5,267 cases and 3,498 controls) were focused on TKA, 41 studies [11, 12, 25, 32, 33, 42–44, 48, 52–54, 62, 64, 67–69, 74, 77, 79, 83, 88, 89, 94, 101–103, 116, 122, 125, 129, 131–133, 138, 139, 143, 144, 148, 150, 152] (2,655 cases and 1,941 controls) on THA, and 17 studies [15, 16, 39, 41, 46, 59, 71, 86, 91, 95, 112, 115, 120, 145, 149, 155, 157] (1,025 cases and 1,035 controls) on other lower limb surgeries. Four articles analyzed both TKA and THA: two of them [79, 88] reported separately VTE complications for TKA and THA and thus were considered in each type of surgery group; conversely, the other two [97, 100] did not report separately VTE complications and thus were not allocated in any group. The administration protocol of TXA included 3 types of administration routes: IV (111 studies, 5,599 cases and 4,980 controls), IA (45 studies, 2,756 cases and 2,495 controls), and oral (7 studies, 712 cases and 362 controls). Studies applying a combination of modalities were included only in the largest group according to the following order: IV>IA>oral. All modalities (dosage amount and bolus versus continuous infusion) and timing (preoperative, intraoperative, or postoperative) of TXA administration were included without further selection.

The quantitative analysis on VTE was first performed and reported on the total of the studies included, then separately for TKA, THA, and other orthopedic surgical procedures of the lower limb. Moreover, a subanalysis for the different administration routes (IV, IA, and oral) with enough available studies reporting VTE was performed.

### 3.1. Total Lower Limb Orthopedic Surgical Procedures

Among 140 high-level RCTs, including 9,067 patients receiving TXA and 6,592 controls, no significant difference in the occurrence of thromboembolic complications between the two groups was detected, with a total VTE rate of 2.4% and 2.8% for TXA and controls, respectively (OR 0.91 [95% CI 0.76-1.09]; *p* = 0.291). With regard to the administration route, the 111 RCTs [12, 15, 16, 21, 22, 26, 30–35, 37–40, 42–45, 47–55, 57, 59–64, 66–68, 70–87, 89–95, 97–100, 103, 105–150, 152–157] comparing IV TXA versus controls, on 5,599 and 4,980 patients, respectively, reported a rate of VTE equal to 2.7% and 2.6%, respectively (OR 0.96 [95% CI 0.77-1.20]; *p* = 0.709). The 45 RCTs [11, 21, 24, 25, 27, 28, 36, 41, 45–47, 56, 58, 65, 69, 75, 79–81, 88, 96, 99, 101, 102, 104–108, 117, 118, 123, 126, 127, 129–132, 134–137, 139, 140, 144] comparing IA TXA administration (2,756 patients) and controls (2,495 patients) reported a rate of VTE of 2.3% and 2.8%, respectively (OR 0.84 [95% CI 0.60-1.17]; *p* = 0.307). Only 7 studies [23, 124, 125, 136, 143, 147, 151] compared VTE events between oral TXA (712 patients) and controls (362 patients), reporting a rate of 1.0% and 2.8%, respectively (OR 0.66 [95% CI 0.28-1.58]; *p* = 0.391).

### 3.2. Total Knee Arthroplasty

**Figure 3:**
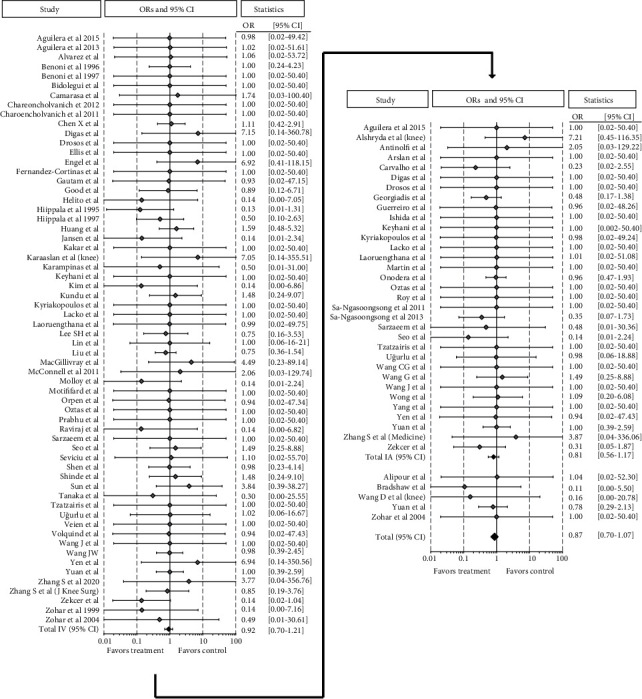
Forest plot of the 82 studies comparing the VTE rate between the TXA group and the control group for TKA. Point estimates of the weighted odds ratios for each study are represented by squares, and the 95% CIs are represented by horizontal bars. The summary odds ratio is represented by a black diamond.

The analysis of the 82 RCTs on TKA showed a total of 5,267 patients belonging to the TXA group with 164 VTE and 3,498 patients of the control group with 135 VTE, without any significant difference in terms of VTE complications, being 3.1% and 3.9%, respectively (OR 0.86 [95% CI 0.70-1.07]; *p* = 0.189). Among 62 RCTs comparing 3,122 TXA administered IV to 2,687 controls, the number of VTE complications was 105 and 90, respectively, with a corresponding equal VTE rate of 3.4% (OR 0.92 [95% CI 0.70-1.21]; *p* = 0.549). Analogously, the meta-analysis of the 33 studies about IA TXA administration showed no significant difference for the VTE rate (1,713 out of 3,178 patients) compared to the control group (1,465 out of 3,178 patients), being 3.0% and 4.1%, respectively (OR 0.81 [95% CI 0.56-1.17]; *p* = 0.268) (Figure 3).

### 3.3. Total Hip Arthroplasty

**Figure 4:**
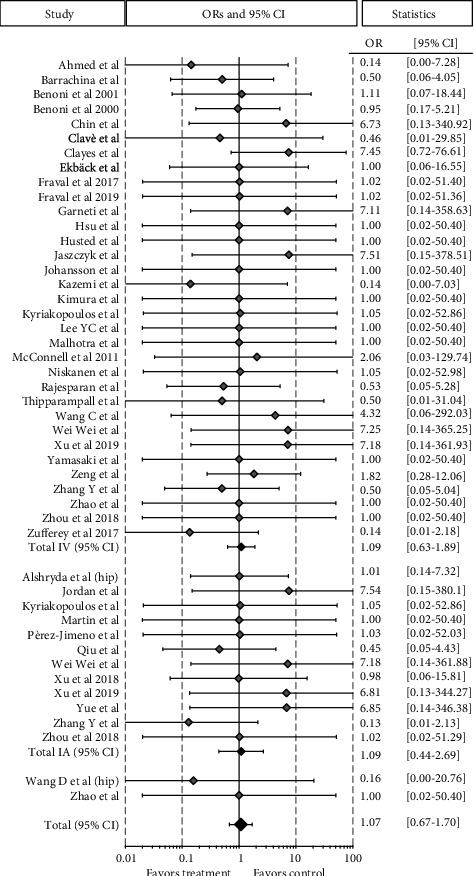
Forest plot of the 41 studies comparing the VTE rate between the TXA group and the control group for THA. Point estimates of the weighted odds ratios for each study are represented by squares, and the 95% CIs are represented by horizontal bars. The summary odds ratio is represented by a black diamond.

Regarding THA, a total of 41 studies with 2,655 patients belonging to the TXA group and 1,941 patients to the control group reported 30 and 21 VTE, respectively, without any significant difference in terms of VTE complications, being 1.1% in both groups (OR 1.07 [95% CI 0.67-1.70]; *p* = 0.769). Among the 33 RCTs comparing TXA (1,583 patients) administered IV to controls (1,389 patients), the rates of VTE were 1.4% and 1.2%, respectively (OR 1.09 [95% CI 0.63-1.89]; *p* = 0.749). Similarly, the meta-analysis of 12 studies on IA TXA showed no significant difference in the VTE rate compared to controls, being 1.0% and 0.9%, respectively (OR 1.09 [95% CI 0.44-2.68]; *p* = 0.857) (Figure 4).

### 3.4. Other Lower Limb Orthopedic Surgical Procedures

**Figure 5:**
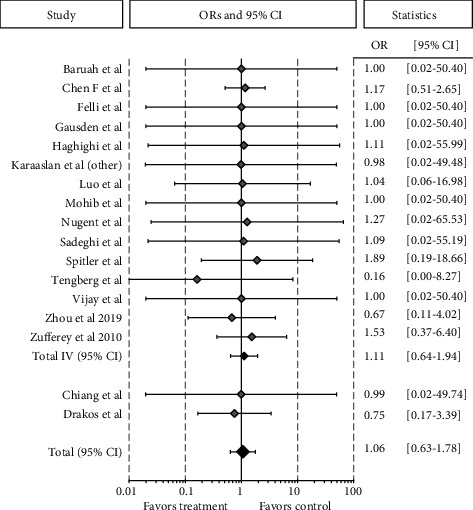
Forest plot of the 17 studies comparing the VTE rate between the TXA group and the control group for other orthopedic lower limb surgical procedures. Point estimates of the weighted odds ratios for each study are represented by squares, and the 95% CIs are represented by horizontal bars. The summary odds ratio is represented by a black diamond.

Among the 17 RCTs on the patients undergoing other lower limb surgical procedures, including intertrochanteric fractures (13 studies, 763 cases and 771 controls), meniscectomies (1 study, 18 cases and 23 controls), and ACL reconstructions (3 studies, 244 cases and 241 controls), 1,025 patients were included in the TXA group and 1,035 in the control group, reporting a similar total rate of VTE of 2.6% and 2.4%, respectively (OR 1.06 [95% CI 0.63-1.77]; *p* = 0.827). Regarding the IV administration group, the meta-analysis of 15 studies had a similar rate of VTE in the 774 patients with TXA compared to 786 controls, being 3.1% and 2.7%, respectively (OR 1.11 [95% CI 0.64-1.93]; *p* = 0.712) (Figure 5).

## 4. Discussion

The main finding of this RCT meta-analysis was that TXA did not increase the risk of VTE complications in patients undergoing lower limb orthopedic surgery. This finding remained consistent for patients undergoing both THA and TKA and other lower limb surgical procedures, as well as for different TXA administration routes (IV, IA, and oral).

TXA was introduced in the clinical practice as a synthetic antifibrinolytic agent aiming at reducing intra- and postoperative blood loss. This represents one of the most common complications associated with major orthopedic surgeries resulting in increased impairment of functional ability, longer hospitalization, and increased morbidity and mortality [158]. Allogenic transfusions are often used to address postoperative anemia and its detrimental consequences, but they are not free from serious adverse events. Accordingly, TXA has been increasingly employed in recent years in replacement surgery of the lower limb joints, and now plenty of high-level studies regarding RCTs and meta-analyses have been published, demonstrating its effectiveness in reducing blood loss and consequently the need for postoperative transfusions [159, 160]. Among these, in 2015, Wu et al. [159] published a meta-analysis on 34 studies and reported that IV or IA use of TXA significantly reduced the postoperative blood loss, hemoglobin decrease, and transfusion rate after primary TKA, though without reducing intraoperative blood loss. Similarly, in the same year, Wei and Liu [160] analyzed 39 trials, confirming that the administration of TXA significantly reduced blood loss and the need for allogeneic blood transfusion. In light of such promising results, the use of TXA has been recently extended also to other lower limb procedures, such as ACLR and meniscectomies, where the aim of TXA, in addition to reducing perioperative blood loss, is to improve early functional recovery. Promising results have been shown also for these indications, with a significant reduction of hemarthrosis and the amount of suction drainage blood volume, as well as improved ROM and quadriceps strength and a lower rate of fever during the first 2 weeks after surgery [15]. Despite the positive findings of these recent attempts to broaden the application of TXA in orthopedic surgery, there are still concerns about its use among orthopedic surgeons and anesthesiologists regarding its safety, in particular the possible increased risk of VTE [17, 18].

In the current study, a meta-analysis was performed to quantify the complication rate reported for TXA compared to controls in high-level trials. In these studies, TXA was used for the intra- and postoperative blood loss management in TKA and THA and also in other lower limb surgical procedures, such as ACLR, meniscectomies, and intertrochanteric fractures. Based on this meta-analysis of 140 RCTs, including 15,659 patients, no significant differences were found in the occurrence of VTE between TXA and controls, with a total rate of 2.4% and 2.8%, respectively. Furthermore, included patients were grouped according to the type of surgery (TKA, THA, and other procedures) and to the method of administration (IV, IA, and oral) in order to investigate possible differences in the complication risk in the different subgroups. The subanalysis confirmed no significant differences in terms of the VTE rate, demonstrating the safety of TXA administration for all these surgical procedures regardless of the administration route, either IV, IA, or oral.

Interestingly, the rate of VTE observed in THA, close to 1%, was lower compared to the 3% reported in TKA, and this difference was consistent in both the TXA and control groups. A possible explanation of this lower VTE rate in THA compared to TKA could be traced back to different rehabilitation protocols with earlier weight bearing and the different applications of cement and tourniquet [161–163]. Nevertheless, a strong statistical methodology could be applied to the selected papers only to detect any possible differences between TXA and controls in the different procedures, while it was not possible to perform an indirect comparative analysis of different subgroups according to the specific surgery. Similarly, it was not possible to compare subgroups differing in terms of administration routes within TXA and control groups. However, this aspect has been already investigated in other studies, which have suggested superior safety of topical application of TXA compared with IV application of TXA, based on the finding that topically applied TXA results in a 90% reduction in plasma concentration compared to IV [164]. What was instead proven with robust statistical analysis of high-level trials, all directly comparing TXA and control groups, was that both the IV administration and the IA administration of TXA did not increase the risk of thromboembolic events compared to controls, regardless of the joint or the surgery considered.

While many literature analysis attempts were focused on the effects of TXA in terms of blood loss reduction, only a few studies specifically addressed the possible complications [165, 166]. Fillingham et al. [165] performed a meta-analysis on 78 studies focusing only on joint replacement procedures, including patients exclusively undergoing joint arthroplasty, published up to 2017, reporting that TXA was not associated with an increased risk of VTE also in “high-risk” patients ASA ≥ 3 undergoing TKA. Likewise, in the same year, Franchini et al. [166] concluded that TXA is a safe pharmacological treatment after a meta-analysis on 73 RCTs focused only on IV administration in patients undergoing joint replacements or hip fracture management.

The current analysis included the latest high-level evidence quantifying the VTE risks considering all types of lower limb orthopedic surgeries and all TXA administration routes, not considered in the previous studies, which allowed to further corroborate the TXA safety and extend the interpretation of existing data to a broader scenario of clinical applications. This is of particular importance since the complications of each application should be weighted with the respective expected benefits. In fact, while joint replacement procedures entail high risks of blood loss, which justifies the potential risk of using drugs to reduce blood loss, an increased VTE risk would be less justified in other procedures, where the expected benefit is mainly in terms of faster recovery. In this light, the results of the meta-analysis about patients undergoing ACLR, intramedullary nailing for femoral fractures and meniscectomies, are of importance. In particular, the results confirm no difference in terms of the VTE rate for patients treated with TXA compared to controls, thus supporting its use also for these new indications, which are increasingly explored in both the literature and the clinical practice.

The large number of high-level trials included in this meta-analysis assures the most reliable methodology to synthesize the scientific evidence about complications related to the use of TXA in lower limb orthopedic surgeries. However, this study still presents several limitations. First, it must be underlined that the included studies are heterogeneous with regard to TXA dosing and timing of administration. Moreover, different VTE screening methods were applied in the different studies, and also antithrombotic prophylaxis regimens were not consistent among the included studies. On the other side, the same methods were used within each RCT. Moreover, other studies demonstrated no effect on the risk of VTE in patients under antithrombotic treatment who received TXA during surgery, regardless of the type of antithrombotic prophylaxis, thus not affecting the conclusions of this study [130, 165]. Another limitation may be represented by the analysis only of VTE complications, although this focus was necessary due to the available data. In fact, albeit including a large number of patients, the low frequency of other complications, such as infections, made it not possible to evaluate and compare them. Nevertheless, previous studies did not suggest a higher infection risk when TXA is applied, and in fact, TXA could rather reduce this kind of complication since it was proved that the infection risk is directly correlated with the number of allogeneic blood transfusions administered [167, 168]. Further studies and large registry analysis should investigate deeper these aspects. Finally, the validity of the results regarding the safety of TXA is limited to the patient categories defined by the inclusion and exclusion criteria used in the published literature. In fact, due to concerns about the administration of TXA in high-risk patients, such as those with a history of thromboembolic and ischemic events as well as vascular stents, these types of patients were commonly excluded from RCTs. However, a recent study [165] suggested that patients with ASA ≥ 3 do not carry an increased risk of VTE with TXA administration, demonstrating the possibility to apply it also in such challenging conditions.

Overall, this study not only confirms the safety of TXA but also underlines the need to standardize the administration protocol (dosage, timing, and duration of administration) since great variability exists in the literature. This is particularly important also in consideration of the heterogeneous applications documented in this meta-analysis, which suggests the possibility to broaden the TXA indication also to other surgeries than just joint arthroplasties, including fractures and sport medicine procedures, supporting the safety of using TXA for all lower limb orthopedic surgical procedures.

## 5. Conclusions

This meta-analysis of TXA for lower limb orthopedic surgical procedures showed an increasing interest over time, with most of the articles published in the last 5 years. Moreover, besides the most common applications for joint replacement procedures, TXA use has been recently broadened to other types of surgery. Overall, TXA did not increase the risk of VTE complications. This finding remained consistent for patients undergoing THA, TKA, and other lower limb surgical procedures, regardless of the administration route, thus supporting the safety of using TXA for lower limb orthopedic surgical procedures.

## Data Availability

The data generated during the current study are available from the corresponding author upon reasonable request.
